# Improving Mental Health Through the Regeneration of Deprived Neighborhoods: A Natural Experiment

**DOI:** 10.1093/aje/kwx086

**Published:** 2017-07-11

**Authors:** James White, Giles Greene, Daniel Farewell, Frank Dunstan, Sarah Rodgers, Ronan A. Lyons, Ioan Humphreys, Ann John, Chris Webster, Ceri J. Phillips, David Fone

**Keywords:** health inequalities, mental health, neighborhood, nonrandomized controlled trials, quasi-experimental studies, residence characteristics

## Abstract

Neighborhood-level interventions provide an opportunity to better understand the impact of neighborhoods on health. In 2001, the Welsh Government, United Kingdom, funded Communities First, a program of neighborhood regeneration delivered to the 100 most deprived of the 881 electoral wards in Wales. In this study, we examined the association between neighborhood regeneration and mental health. Information on regeneration activities in 35 intervention areas (*n* = 4,197 subjects) and 75 control areas (*n* = 6,695 subjects) was linked to data on mental health from a cohort study with assessments made in 2001 (before regeneration) and 2008 (after regeneration). Propensity score matching was used to estimate the change in mental health in intervention neighborhoods versus control neighborhoods. Baseline differences between intervention and control areas were of similar magnitude as produced by paired randomization of neighborhoods. Regeneration was associated with an improvement in the mental health of residents in intervention areas compared with control neighborhoods (β = 1.54, 95% confidence interval: 0.50, 2.59), suggesting a reduction in socioeconomic inequalities in mental health. There was a dose-response relationship between length of residence in regeneration neighborhoods and improvements in mental health (*P*-trend = 0.05). These results show that targeted regeneration of deprived neighborhoods can improve mental health.

A series of studies has shown that poor mental health is more common among residents of low-quality housing (1, 2), less desirable neighborhoods (e.g., those with litter, graffiti, and few street lights) (3, 4), and socioeconomically deprived areas (5, 6). Support for these associations has not been universal; all have shown marked attenuation after individual- or household-level measures of socioeconomic disadvantage have been accounted for (1–6). If the association between poor mental health and the quality of the neighborhood environment were causal, this would suggest that neighborhood regeneration could help to reduce inequalities in mental health.

Large regeneration projects are common in the United Kingdom; examples include New Deal for Communities (7) and Go Well (8). Regeneration projects also exist elsewhere, including Empowerment Zones in the United States (9) and the URBAN Community Initiative in mainland Europe (10). Because low-quality housing and neighborhoods (1–3), as well as the wider social, economic, and environmental determinants of poor health, are concentrated in the most deprived areas (4–6), policy-makers often target deprived neighborhoods for regeneration with the expectation that socioeconomic inequality will be reduced and population health will improve. The hypothesized mechanisms through which neighborhood regeneration might improve mental health includes improving physical health through housing improvements (11), increasing social capital and support by establishing community groups (12), enhancing feelings of control and empowerment (12), and reducing crime by enhancing neighborhood security (13).

There have been few studies on the relationship between targeted neighborhood regeneration and mental health. Of the controlled evaluations carried out to date, the New Deal for Communities project, an areawide regeneration scheme in England, United Kingdom, found that regeneration was not associated with change in mental health in comparison with randomly sampled residents from noncontiguous comparator areas (14, 15) or Health Survey for England participants with similar area-level deprivation (16). In the smaller Single Regeneration Budget program, based in Manchester, United Kingdom, no difference in mental health scores was found between residents of intervention wards and neighboring control wards (17). In contrast, the Go Well regeneration program in Glasgow, Scotland, United Kingdom, found that residents of areas receiving housing improvements reported an improvement in mental health in comparison with residents in control areas (18). The improvement was also greater for the residents of areas that received more than £10,000 (US$16,200 at the midyear 2011 currency exchange rate) in investment per household than for residents of areas that received less than £5,000 ($8,100), suggesting a relatively high threshold in the investment required to narrow inequalities (19). Those studies, however, lack detailed assessments of individual- and household-level socioeconomic disadvantage, such that it is unclear whether intervention and comparison groups were adequately matched on socioeconomic factors that are closely associated with poor mental health.

“Communities First” is an areawide socioeconomic regeneration program implemented in deprived neighborhoods in Wales, United Kingdom (19). We exploited an opportunity to evaluate a natural experiment, using data on neighborhoods which did and did not receive regeneration linked to an electronic record-linked prospective cohort study, the Caerphilly Health and Social Needs Electronic Cohort Study (eCATALyST) (20). Previous analyses of eCATALyST found that Communities First was delivered in an area with substantial socioeconomic inequalities in mental health (5). We therefore used propensity score matching to reduce the imbalance in socioeconomic disadvantage across intervention and control areas brought about by nonrandom targeted allocation (20). We tested the hypothesis that the Communities First regeneration program would be associated with a reduction in mental health inequalities.

## METHODS

The protocol for this prospective controlled quasi-experimental study or natural experiment has been published previously (21). We linked data from 3 sources for this study: regeneration data from the Caerphilly County Borough Council (Caerphilly, Wales), the eCATALyST study (22), and the Secure Anonymised Information Linkage (SAIL) Databank (23). The eCATALyST study received ethical approval for the baseline population survey (2001) from the former Gwent Local Research Ethics Committee and for the follow-up survey (2008) from the South East Wales Research Ethics Committee, Panel C. The linking of data sets received approval from the SAIL Information Governance Review Panel at Swansea University (Swansea, Wales).

### Setting

Caerphilly County Borough, located in southeastern Wales, United Kingdom, is a local government unitary authority with a 2001 census population of 169,519, rising to 180,164 in the latest midyear estimate for 2015 (24). In 2001, the year in which the Communities First regeneration program was initiated, there were 22 local authorities in Wales, 881 electoral wards, and 1,896 Lower Layer Super Output Areas (LSOAs) defined in the United Kingdom Census (25). Employment in Caerphilly County Borough has historically been dominated by the coal industry. By 2001, the borough had a higher rate of unemployment than the Welsh average (5.4% vs. 8.5%) and a higher percentage of public housing than the Welsh average (17.1% vs. 13.7%) (26). In the 2000 Welsh Index of Multiple Deprivation (WIMD), a small-area-based measure of deprivation based on residents’ income, employment status, education, housing, health, and geographical access to services, it ranked fourth out of 22 local authorities in Wales in the proportion of LSOAs that were in the 10% most deprived (27). Although intervention in the Communities First program was allocated at the electoral ward level (average population 5,500), we used LSOAs (average population 1,630) as our measure of small-area geography, since they are smaller, more homogeneous, and nested within wards.

### Intervention

Communities First is a Welsh Government program of areawide socioeconomic regeneration. It is targeted at the 100 most deprived of the 881 electoral wards in Wales, according to the 2000 WIMD (27). An extra 46 wards were added in 2005 following a change in rankings when the WIMD was updated. In 2001, each of the 22 local authorities in Wales received funding to establish community multiagency partnership boards which included residents of Communities First areas, the police, the United Kingdom National Health Service, local authorities, and housing providers. Partnership boards conferred with residents to identify what regeneration activities were needed. Once a candidate project was identified, a wider consultation with residents was held; if residents agreed that the project was a priority, an application was made to a potential funder, such as a charity, local government, or private industry. In some cases, funding was provided by the partnership board (28). Communities First was reorganized in 2012, when the ranking of wards changed following publication of the 2011 WIMD. In February 2017, the Welsh Government announced that funding for Communities First would be phased out by March 2018.

We classified the projects delivered into 48 different types of intervention nested within 6 domains of regeneration. We used text descriptions and geographical locations to classify regeneration activities according to a scheme informed by a similar intervention, New Deal for Communities (29). Examples of funded activities in the 6 domains included those designed to address community needs regarding: 1) *crime*—installing street lighting; 2) *education*—providing teaching assistants; 3) *health—*providing sports equipment; 4) *housing and physical environment*—conducting housing maintenance and repairs and redeveloping wasteland; 5) *vocational training and business support*—providing computer skills training to the unemployed; and 6) *community*—building community facilities (see Web Figures 1 and 2 and Web Tables 1 and 2, available at https://academic.oup.com/aje/, for the full classification scheme and additional examples).

### Data sources

#### The eCATALyST study

The eCATALyST study was a prospective cohort study of adult residents of Caerphilly County Borough, Wales, United Kingdom (22). Briefly, in 2001, a stratified random sample of 22,236 persons aged 18 years or more was sent a baseline postal questionnaire survey, and 10,892 people responded. In 2008 the survey was repeated, sampling the 9,551 participants who still resided in the borough. Of these, 4,426 provided a valid mental health score. The patterns of response in 2001 and 2008 showed underrepresentation of younger age groups, particularly men, but no discernable bias by 1991 Townsend area-level deprivation scores (22, 30).

#### The Caerphilly County Borough Council

Council records were used to identify the LSOAs within Caerphilly County Borough that received Communities First funding (intervention areas) and those LSOAs which did not (control areas) between 2001 and 2008.

#### The SAIL Databank

The SAIL Databank is a privacy-protecting digital environment that enables multiple data sets to be pseudonymized and then brought together for analysis. It contains the Welsh Demographic Service (a list of the addresses of all patients registered with cost-free general medical practices in Wales between 1998 and 2012) and clinical data (23). The date of every update for each individual's record is held. The Welsh Demographic Service was used to track migration and calculate length of residence from May 31, 2001, to January 1, 2009, the dates of the eCATALyST surveys. We also used data held in SAIL on mental health symptoms, diagnoses, and treatments recorded in 11 of the 29 general medical practices in Caerphilly County Borough to impute missing eCATALyST data.

### Variables

#### Mental health

Mental health was assessed using the 5-item Mental Health Inventory (MHI-5) included in the Short Form 36 (version 2) scale (31), measured in the 2001 and 2008 waves of eCATALyST. The validity and reliability of the MHI-5 in general population samples are well-established (31), and it is effective at screening for mood and anxiety disorders identified using the Diagnostic Interview Schedule (32). Respondents can achieve a total score within a range of 5–25, which we transformed to a 0–100 scale (33). The primary outcome was change in mental health score (wave 2 minus wave 1), with positive values indicating an improvement in mental health.

#### Demographic and socioeconomic factors

The eCATALyST database contained information on a number of individual, household, and area-level demographic and socioeconomic variables (5). These included age (in 10-year bands), sex, United Kingdom Registrar General social class (I and II (most advantaged), IIINM, IIIM, IV and V, or other), employment status (employed, unemployed, full-time student/government training scheme, looking after the home or children full-time/long-term caregiver, permanently unable to work due to illness or disability, or retired), housing tenure (owner-occupier or not owner-occupier), council tax valuation band of property values (A (lowest values), B, C, D, E, or F-H (highest values)) (34), poverty (defined as gross household income <£10,000 per annum ($14,100 at midyear 2001 currency exchange rate), which equated to 60% of median income after housing costs in 2001 (the United Kingdom definition of household poverty)), 2005 WIMD at the LSOA level (35), marital status (married/cohabiting, single, divorced, or widowed), smoking status (daily, occasional, former, or never smoker), tertile of the sum of neighborhood cohesion scales at the individual and LSOA levels (assessing levels of social interaction, trust, and reciprocity within neighborhoods) (36), and physical health, assessed using the Physical Component Summary score of Short Form 36 (range, 0–100) (31).

### Statistical analysis

Unless specifically indicated, all analyses were intention-to-treat and preplanned in accordance with our published protocol (21). We assessed whether there were interactions between allocation status and age group and sex and found that these interactions were not statistically significant. We therefore pooled data for men and women and across age groups.

We estimated the effectiveness of regeneration in reducing inequalities in mental health by matching residents in 35 intervention LSOAs to residents in 75 control LSOAs based on their propensity scores, using information from the 2001 eCATALyST survey (all 4,197 intervention group participants were matched to the same number of control group participants; *n* = 4,197). The variables included in calculation of the propensity score were employment status, housing tenure, council tax band, poverty, and marital status (see Web Appendix 1 for details).

To investigate the different types of interventions, we fitted models in which the binary term for allocation status was replaced with a categorical term for the 6 domains of regeneration (with control areas as the reference category).

#### Missing data

eCATALyST data were weighted to allow for the unequal electoral ward sampling probability and survey nonresponse as a function of age, sex, and 1991 Townsend deprivation scores (30), the latest available measure of area-level deprivation in the Welsh Demographic Service for the whole sampling frame (20). Data on allocation status and covariates were available for 4,426 people who had a mental health score. We used Read codes (37), a system of clinical coding used in general medical practices in the United Kingdom, on mental health symptoms, diagnoses, and treatments as auxiliary data to impute missing postintervention covariate and outcome data in the eCATALyST database (see Web Appendix 2).

Of the 6,466 participants who did not provide complete data in the 2008 eCATALyST survey, 1,504 (23.3%) had a Read code for a symptom, diagnosis, or treatment for a mood or anxiety disorder recorded in general practice. A variable with the absence (coded as 0) or presence (≥1 coded as 1) of a relevant Read code across all years (2001–2008) for all participants was derived. The prediction model comprised all eCATALyST covariates including the presence or absence of any mental health Read codes. We imputed the raw scores for all eCATALyST covariates using multiple imputation by chained equations to generate 20 imputed data sets (each had a final *n* of 10,892), accounting for the hierarchical structure of the data set (individuals nested within LSOAs). Web Figure 1 shows the data sources, numbers of participants linked, and data imputed.

#### Sensitivity analysis

We performed several sensitivity analyses. First, we conducted our analysis in a complete sample of 4,426 participants without any missing data. Second, we replaced a binary term for allocation with tertiles for the frequency of different types of regeneration activities. Third, we examined whether the intention-to-treat principle had caused the association between regeneration and mental health to be underestimated by repeating the analyses after replacing allocation with duration of residence in an intervention area, categorized into quartiles. Fourth, we examined the impact of population migration by including a term in models for whether a participant had moved. Fifth, we repeated the analysis adjusting for covariates included in the propensity score to examine their effect on changes in mental health.

All analyses were performed with Stata, version 13 (StataCorp LP, College Station, Texas), MLwiN, version 2.32 (Centre for Multilevel Modelling, Bristol, United Kingdom), and R (R Foundation for Statistical Computing, Vienna, Austria). The type I error probability was set to 0.05 for all analyses. The reporting of results from this study conforms to the Transparent Reporting of Evaluations with Nonrandomized Designs statement (available at https://academic.oup.com/aje) (38).

## RESULTS

There were 1,500 funded regeneration projects in Caerphilly County Borough during the 7-year follow-up period (2001–2008) at a cost of £82,857,180 ($161,571,501 at midyear 2008 currency exchange rate). Of these projects, over one-half (59.1%; £16,489,716 ($32,154,946)) were classified as community-based projects, with the remaining classified as improvements in housing and the physical environment (22.3%; £55,670,516 ($108,557,506)), projects in educational settings (8.0%; £5,534,839 ($10,792,936)), health improvement (4.7%; £1,321,639 ($2,577,196)), interventions to reduce crime (4.2%; £1,742,129 ($3,397,152)), and the provision of vocational training or business support (1.7%; £2,098,341 ($4,091,765)).

Of the 10,892 participants, 4,197 (38.5%) were located in 35 intervention LSOAs and 6,695 in 75 control LSOAs (see Web Figure 2). The mean length of residence during the study period was 58.8 months (standard deviation, 17.0; range, 0.2–91.0). A total of 208 (5.0%) intervention residents and 160 (2.4%) control residents moved between 2001 and 2008. People with missing eCATALyST postintervention data in 2008 were more likely to be older, to have a semiroutine or routine occupation, to be disabled, to be living in poverty, to be married, and to be a daily smoker but were less likely to be a homeowner (all *P*’s < 0.05).

As would be expected for an intervention delivered on the basis of area-level deprivation, the largest differences between the intervention and control groups were found for employment status, housing tenure, council tax valuation band, and poverty status (Table 1). After matching, the absolute standardized differences were less than 10% for all variables entered into the propensity score, indicating an acceptable match (Table 2) (39).

**Table 1.** Characteristics of Residents of Intervention and Control LSOAs Before (2001) and After (2008) the Introduction of a Neighborhood Regeneration Program (Communities First), Caerphilly County Borough, Wales, United Kingdom

Variable	Intervention LSOAs (*n* = 4,197)					Control LSOAs (*n* = 6,695)				
	2001		2008		Change^a^	2001		2008		Change^a^
	No. of Persons	%	No. of Persons	%		No. of Persons	%	No. of Persons	%	
Mental health^b,^^c^	22.3 (66.6)		19.9 (66.2)		−0.4	20.8 (71.0)		18.3 (70.8)		−0.2
Physical health^b^	13.3 (44.7)		12.2 (43.7)		−1.0	12.5 (47.3)		11.7 (45.7)		−1.6
Neighborhood social cohesion^d^										
Low	1,083	25.8	1,152	27.5	1.7	1,086	27.0	1,876	28.0	1.0
Medium	1,537	36.6	1,319	31.4	−5.2	2,607	38.9	2,328	34.8	−4.1
High	1,577	37.6	1,726	41.1	3.5	2,282	34.1	2,491	37.2	3.1
Social class^e^										
I or II	755	18.0	967	23.0	5.0	1,799	26.8	2,165	32.3	5.5
IIINM	772	18.4	777	18.5	0.1	1,520	22.7	1,457	21.8	−0.9
IIIM	961	22.9	932	22.2	−0.7	1,420	21.2	1,407	21.0	−0.2
IV or V	1,343	32.0	1,398	33.3	1.3	1,603	24.0	1,553	23.2	−0.8
Other	366	8.7	123	2.9	−5.8	353	5.3	113	1.7	−3.6
Employment										
Employed	1,953	46.5	1,799	42.9	−3.6	3,728	55.7	3,340	49.9	−5.8
Unemployed	135	3.2	115	2.7	−0.5	169	2.5	124	1.9	−0.6
Student	62	1.5	25	0.6	−0.9	133	2.0	33	0.5	−1.5
Homemaker or caregiver	406	9.7	284	6.8	−2.9	447	6.7	383	5.7	−1.0
Permanently sick or disabled	719	17.1	586	14.0	−3.1	710	10.6	616	9.2	−1.4
Retired	922	22.0	1,388	33.1	11.1	1,508	22.5	2,199	32.9	10.4
Housing tenure (not an owner-occupier)	1,081	25.8	1,151	27.4	1.6	985	14.7	1,098	16.4	1.7
Council tax valuation band										
A (lowest property values)	1,891	45.1	1,728	41.2	−3.9	688	10.3	482	7.2	−3.1
B	1,568	37.4	1,481	35.3	−2.1	2,874	42.9	2,199	32.9	−10.0
C	376	9.0	508	12.1	3.1	1,535	22.9	1,927	28.8	5.9
D	196	4.7	214	5.1	0.4	821	12.3	1,056	15.8	3.5
E	125	3.0	196	4.7	1.7	483	7.2	674	10.1	2.9
F-H (highest property values)	41	1.0	70	1.7	0.7	294	4.4	357	5.3	0.9
Living in poverty^f^	2,466	58.8	2,123	50.6	−8.2	2,939	43.9	2,600	38.8	−5.1
Smoking status										
Daily smoker	1,160	27.6	875	20.9	−6.7	1,461	21.8	1,136	17.0	−4.8
Occasional smoker	217	5.2	247	5.9	0.7	304	4.5	305	4.6	0.1
Former smoker	979	23.3	1,208	28.8	5.5	1,609	24.0	1,876	28.0	4.0
Never smoker	1,841	43.9	1,867	44.5	0.6	3,321	49.6	3,378	50.5	0.9
Marital status^g^										
Married/cohabiting	2,808	66.9	2,708	64.5	−2.4	4,874	72.8	4,806	71.8	−1.0
Single	678	16.2	559	13.3	−2.9	910	13.6	685	10.2	−3.4
Divorced or separated	404	9.6	469	11.2	1.6	490	7.3	583	8.7	1.4
Widowed	307	7.3	461	11.0	3.7	421	6.3	621	9.3	3.0

Abbreviation: LSOA, Lower Layer Super Output Area.

^a^ Percentage-point change (postintervention (2008) minus preintervention (2001)).

^b^ Values are expressed as mean (standard deviation).

^c^ Measured by means of the 5-item Mental Health Inventory, which has a score range of 5–25 (31). Scores were transformed to a 0–100 scale using a standard linear transformation.

^d^ Cutpoints on the social cohesion scale: low = 0–16; medium = 17–31; high = 32–40.

^e^ United Kingdom Registrar General social class: I = professional occupations; II = managerial and technical occupations; IIINM = skilled, nonmanual occupations; IIIM = skilled manual occupations; IV = partly unskilled occupations; V = unskilled occupations; other = other occupations, including student, homemaker, or unemployed.

^f^ Poverty was defined as gross household income <£10,000 per annum ($14,100 at midyear 2001 currency exchange rate), which equated to 60% of median income after housing costs in 2001 (the United Kingdom definition of household poverty).

^g^ Marital status at baseline was retrospectively assessed for 2001 in 2008.

**Table 2.** Baseline Demographic and Socioeconomic Characteristics of Residents of Intervention and Control LSOAs Before (2001) and After (2008) Propensity Score Matching, Caerphilly County Borough, Wales, United Kingdom

Variable	Before Matching (*n* = 10,892)					After Matching (*n* = 8,394)				
	Intervention LSOAs (*n* = 4,197)		Control LSOAs (*n* = 6,695)		Standardized Difference^a^	Intervention LSOAs (*n* = 4,197)		Control LSOAs (*n* = 4,197)		Standardized Difference^a^
	No. of Persons	%	No. of Persons	%		No. of Persons	%	No. of Persons	%	
Employment status										
Employed	1,953	46.5	3,728	55.7	18.3	1,953	46.5	1,952	46.5	0.1
Unemployed	135	3.2	169	2.5	4.8	135	3.2	123	3.0	0.1
Student	62	1.5	133	2.0	3.7	62	1.5	62	1.5	0.1
Homemaker or caregiver	406	9.7	447	6.7	10.5	412	9.7	412	9.8	0.1
Permanently sick or disabled	719	17.1	710	10.6	18.6	720	17.1	720	17.1	0.0
Retired	922	22.0	1,508	22.5	1.2	928	22.0	928	22.0	0.0
Housing tenure (not an owner-occupier)	1,081	25.8	985	14.7	27.5	1,081	25.7	1,078	25.8	0.0
Council tax valuation band										
A (lowest property values)	1,891	45.1	688	10.3	84.7	1,891	45.3	1,891	45.3	0.0
B	1,568	37.4	2,874	42.9	12.0	1,568	37.2	1,568	37.2	0.0
C	376	9.0	1,535	22.9	38.3	376	9.0	378	9.2	0.0
D	196	4.7	821	12.3	27.7	196	4.7	194	4.6	0.0
E	125	3.0	483	7.2	20.5	125	2.9	124	2.8	0.0
F-H (highest property values)	41	1.0	294	4.4	20.0	41	1.0	42	1.0	0.0
Living in poverty^b^	2,466	58.8	2,939	43.9	30.1	2,466	58.8	2,478	59.0	0.0
Marital status										
Married/cohabiting	2,808	66.9	4,874	72.8	13.1	2,808	66.9	2,836	67.1	0.1
Single	678	16.2	910	13.6	7.8	678	16.2	668	15.9	0.0
Divorced or separated	404	9.6	490	7.3	8.5	404	9.6	407	9.7	0.0
Widowed	307	7.3	421	6.3	3.4	307	7.3	307	7.3	0.1

Abbreviation: LSOA, Lower Layer Super Output Area.

^a^ Standardized difference was calculated as $d=\frac{100\times (\bar{x}\;\mathrm{treatment}-\overset{¯}{\overset{¯}{x}}\;\mathrm{control})}{\sqrt{\frac{s^{2}\;\mathrm{treatment}+s^{2}\;\mathrm{control}}{2}}}$.

^b^ Poverty was defined as gross household income <£10,000 per annum ($14,100 at midyear 2001 currency exchange rate), which equated to 60% of median income after housing costs in 2001 (the United Kingdom definition of household poverty).

The descriptive data showed a reduction in the mental health of all participants over the course of the study period. The mean intervention group mental health (MHI-5) score declined by 0.4 points, with a corresponding mean reduction of 0.2 points in the control group (Table 1). After propensity score matching, targeted regeneration was associated with an improvement in the mental health of intervention group residents compared with control group residents (β = 1.54, 95% confidence interval: 0.50, 2.59), suggesting that regeneration narrowed mental health inequalities.

The results of the sensitivity analyses are shown in Web Appendix 3. We found essentially the same pattern of results in our complete-case and imputed samples (Web Appendix 3, Web Table 3). We found similar effect sizes but no significant differences when type of intervention was examined (Web Appendix 3, Web Table 4). We found evidence of a dose-response association such that the longer the duration of residence in an intervention area the greater the improvement in mental health (*P* for trend = 0.05; Web Appendix 3, Web Table 5). The change in mental health was comparable in the models which did and did not exclude migrants from intervention and control areas (Web Appendix 3, Web Table 6). In the covariate-adjusted analysis, being an owner-occupier, being sick or disabled, or being retired was associated with an improvement in mental health (Web Appendix 3, Web Table 7).

## DISCUSSION

We estimated the impact of socioeconomic regeneration on mental health in a natural experiment by linking data from a regeneration program delivered to deprived communities to data from a prospective cohort study that collected data both before and after intervention. We found that the targeted regeneration of deprived neighborhoods narrowed inequalities in mental health, and we found some evidence for a dose-response association between length of residence in a regeneration area during the study period and improvements in mental health.

In propensity-score-matched analyses, regeneration in neighborhoods that had the greatest concentration of socioeconomic disadvantage was associated with a small improvement in the mental health of intervention-area residents compared with control-area residents; thus, inequalities in mental health narrowed. The effect size was equivalent to 1 out of every 3 intervention group residents increasing their response on the MHI-5 scale by 1 category (e.g., from “most of the time” to “all of the time”) or 7% of a standard deviation on the MHI-5 scale. This narrowing of inequality was found at relatively low cost, with the average cost of a community project being £18,590 ($36,251 at midyear 2008 currency exchange rate) and the average cost of a housing project being £166,180 ($324,051). The results contrast with those from most of the prospective controlled evaluations that found no association between regeneration in deprived neighborhoods and changes in mental health (14–17), but they replicate the narrowing of mental health inequalities found in the Go Well regeneration project (19). These inconsistencies may be attributed to differences in the effectiveness of regeneration, differences in sampling of areas or residents, or our use of propensity score matching.

The main strength of this study was the availability of data from detailed pre- and postintervention assessments of socioeconomic disadvantage carried out among residents who participated in a prospective cohort study. The SAIL system enabled clinical records, migration, and duration of residence to be assessed. Our use of propensity score matching allowed us to account for the baseline differences in mental health scores by balancing several characteristics of participants (such as employment status) and households (poverty status) between intervention and control areas. Another concern was selective migration (40), but we found little difference in estimates for those who had and had not moved out of intervention areas.

The main limitation of this study was that a direct measure of individual exposure could not be captured by the data. Although this is common to most evaluations of neighborhood-level interventions, not all residents of intervention areas will have been exposed, and residents of control neighborhoods may have been exposed as people crossed boundaries. This would have acted to underestimate the effect size. A further limitation was loss to follow-up. Although the study had sufficient statistical power to detect a difference across intervention and control areas (21), it is likely that the subgroup analysis of the types of regeneration activities was underpowered, increasing the risk of type II error. The use of auxiliary information on mental health symptoms, diagnoses, and treatments meant that for 22.5% of participants with missing cohort data, relevant information on mental health was available from general practice to improve the imputation model.

The information on intervention subtype relied heavily on the accuracy of Caerphilly County Borough Council records, and it is possible that not all funded activities were recorded or not all those funded through another source were recorded. Our reliance on text descriptions of projects meant that the type of intervention could have been misclassified, which could have biased the subgroup analysis towards the null. A general limitation with propensity score analysis is that it can only balance confounders included in the model; thus, our estimates may have been different if other important variables had been included. The estimates derived from propensity scores can be comparable to those from adjustment in regression (41), particularly when using propensity scores as a covariate, but less so when matching participants as we did.

This study has wider implications for the methods used to evaluate nonrandomized targeted interventions. Given the enormous amounts of money spent on areawide regeneration in the United States, the United Kingdom, and Europe and the paucity of evidence on its effectiveness, randomized allocation of areas may be both the fairest means of allocation and the best method of enabling robust evaluation to inform decisions on continued support or disinvestment (7–10). We found that propensity score matching was able to balance intervention and control groups for known confounding factors in an area with socioeconomic inequalities in mental health. In future studies, investigators should compare effect-size estimates from propensity-score-matched analyses with those from randomized trials to examine the value of this analytical approach.

The regeneration program we evaluated, Communities First, is unique in that community residents, rather than local councils or governments, identified areas to be regenerated. The policy implication of this finding is that targeted regeneration directed by the residents of deprived urban communities may help to reduce inequalities in mental health.

## Supplementary Material

Web Material
