# Supplementary material for: Improving Mental Health Through the Regeneration of Deprived Neighborhoods: A Natural Experiment
Source: Am J Epidemiol. 2017 Jul 11;186(4):473–80. doi: 10.1093/aje/kwx086 (PMC5860549; doi:10.1093/aje/kwx086)
Supplement: Web Material [file kwx086whitewebmaterialfinal.pdf]

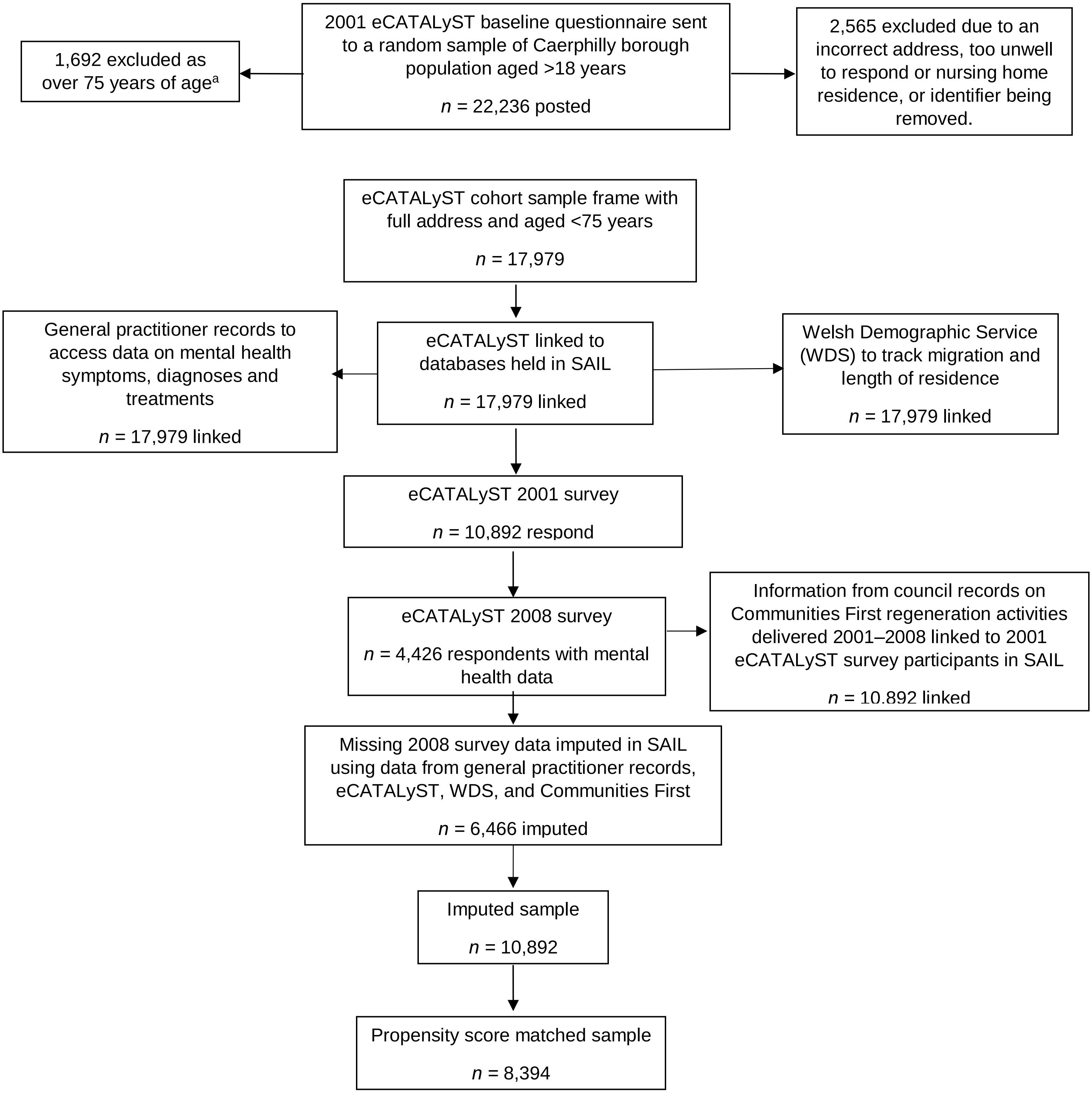

Web Figure 1. Flow Diagram of Participation in the Caerphilly Health and Social Needs Electronic Cohort Study (eCATALyST), Record Linkages, and Imputation.

[a] Restricted to residents aged <75 years on May 31, 2001, because the 5-item Mental Health Inventory (MHI-5) outcome measure scale is less reliable in United Kingdom elderly populations (1, 2).

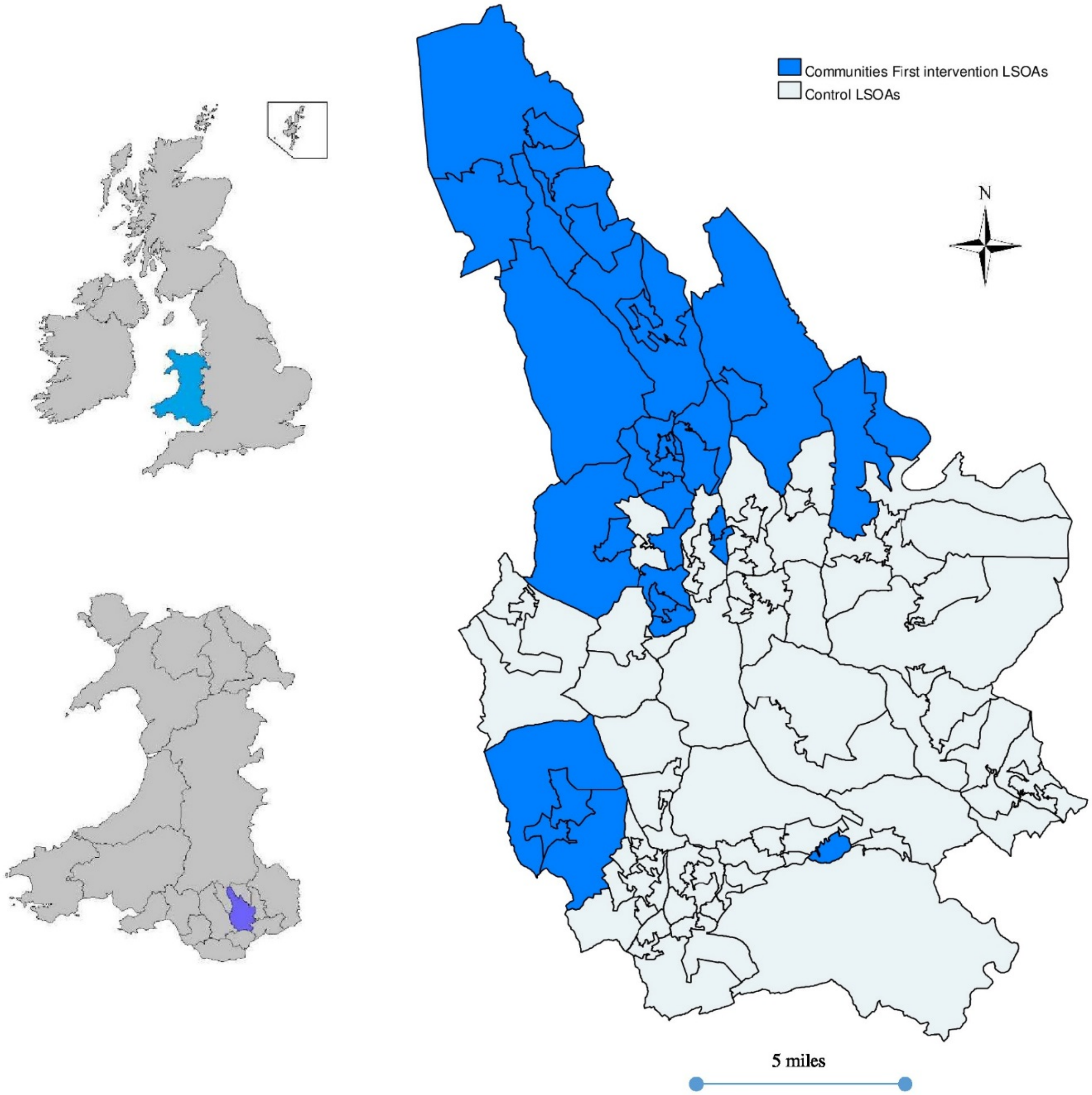

Web Figure 2. The 35 Communities First Intervention Lower Layer Super Output Areas (LSOAs) and 75 Control LSOAs in Caerphilly County Borough, Wales, United Kingdom.

Web Table 1. Classification of Communities First Regeneration Activities and the Number (%) of Activities Funded, Caerphilly County Borough, Wales, United Kingdom, 2001–2008

Subtheme	No.	% Within Subtheme
Crime		
Increase police numbers	5	7.9
CCTV	26	41.3
Domestic security project	1	1.6
Street lighting	27	42.9
Nondomestic security project (e.g. install intruder alarm in a community centre)	4	6.3
Education		
Educational support (e.g. advertise adult learners event)	25	20.8
Family learning/support (e.g. staff parenting class)	8	6.7
After school/breakfast/holiday club	36	30.0
Basic information and communications technology (ICT) skills	1	0.8
School maintenance, new builds/extensions	12	10
Early learning / Surestart	15	12.5
Theme development / staff	2	1.7
Educational facility	5	4.2
School / related programme (e.g. provide sports equipment)	16	13.3
Health		
Health improvement/promotion (e.g. dancing lessons)	23	32.9
Drug/alcohol abuse worker/project	2	2.8
Health services ability/assessment (e.g. arthritis support)	9	12.9
Food and nutrition project (e.g. healthy eating classes)	3	4.3
Older peoples project (e.g. widows club, over-60's lunch club)	25	35.7
Healthy living centre (e.g. staffing of health centre)	8	11.4
Housing and physical environment		
Environmental improvement project (e.g. woodland conservation)	112	33.4
Physical redevelopment (e.g. resurface car park)	32	9.5
Green, open spaces and parks (e.g. redevelop disused land into parkland)	34	10.3
Housing maintenance, and building (e.g. replacing an old boiler)	74	22.1
Transport - public, community and related (e.g. bus hire)	14	4.1
Play grounds/ areas (e.g. install new play equipment)	30	9.0
Housing management	7	2.1
Property acquisition and demolition (e.g. demolition of public housing)	7	2.1
Highways/road/traffic calming (e.g. build new road, install cycle pathways)	25	7.4
Vocational training or business support		
Business support and development (e.g. loans)	17	68.0
New business/self-employment	1	4.0
Training schemes/advice	1	4.0
Skills training	4	16.0
ICT project to improve employability	2	8.0

Community		
Marketing and communications	4	0.5
Community participation (e.g. fund community events on health, local history)	172	19.4
Community facility (e.g. building a community centre, fit a new roof)	182	20.5
Leisure project/facility	149	16.8
Theme development/staff	5	0.6
Youth project/staff (e.g. staffing youth clubs)	103	11.6
Funding (e.g. volunteers expenses)	29	3.3
Children and young person's facility/activity	58	6.5
Advice, support or information and or centres	15	1.7
IT infrastructure	22	2.5
Sport or exercise project/facility (e.g. new sports equipment)	112	12.5
New community facilities (e.g. furniture/kitchen)	36	4.1

Web Table 2. Number and Percentage of Communities First Regeneration Activities Funded by Wards and Type, Caerphilly County Borough, Wales, United Kingdom, 2001–2008

Ward	No. of LSOAs	No. of Projects	Crime		Education		Health		Housing		Vocational		Community	
			No.	%	No.	%	No.	%	No.	%	No.	%	No.	%
Aber Valley	4	149	6	4.0	11	7.4	6	4.0	27	18.1	2	1.3	97	65.1
Abertysswg	1	37	3	8.1	3	8.1	3	8.1	6	16.2	0	0.0	22	59.5
Cefn Fforest	2	69	3	4.3	3	4.3	3	4.3	10	14.5	2	2.9	48	69.6
Cefn Hengoed	3	135	2	1.5	23	17.0	9	6.7	10	7.4	3	2.2	88	65.2
Deri	1	67	4	6.0	5	7.5	2	3.0	19	28.4	2	3.0	35	52.2
Fochriw	1	78	6	7.7	5	6.4	8	10.3	26	33.3	2	2.6	31	39.7
Gelligaer and Penybryn	2	81	0	0.0	7	8.6	3	3.7	21	25.9	1	1.2	49	60.5
GlanyNant and Tirybeth	1	30	0	0.0	2	6.7	3	10.0	8	26.7	0	0.0	17	56.7
Graig Y Rhacca	1	110	8	7.3	5	4.5	1	0.9	22	20.0	3	2.7	71	64.5
Bargoed	7	268	8	3.0	22	8.2	17	6.3	36	13.4	5	1.9	180	67.2
New Tredegar	3	131	3	2.3	9	6.9	5	3.8	54	41.2	2	1.5	58	44.3
Pontlottyn	1	57	5	8.8	3	5.3	3	5.3	16	28.1	1	1.8	29	50.9
Rhymney	4	131	6	4.6	6	4.6	6	4.6	45	34.4	0	0.0	68	51.9
Trinant, Pentwyn and Cwmnantgwynt	2	74	4	5.4	6	8.1	1	1.4	19	25.7	1	1.4	43	58.1
Upper Sirhowy Valley	2	83	5	6.0	10	12.0	0	0.0	16	19.3	1	1.2	51	61.4
**Total**	**35**	**1,500**	**63**	**4.2**	**120**	**8.0**	**70**	**4.7**	**335**	**22.3**	**25**	**1.7**	**887**	**59.1**

Web Appendix 1: Propensity score analysis

We determined a propensity score for residence in an intervention area using a logistic regression model (1, (4), (5). A key assumption of nonexperimental studies is a strongly ignorable treatment assignment, whereby the treatment allocation is independent of the observed and unobserved covariates (6). To satisfy the assumption of ignorability, we only included covariates from the 2001 eCATALyST survey into the propensity score calculation that were associated (at the $P<0.05$ level) with both allocation status and change in mental health (7). These variables were: employment status, housing tenure, council tax band, poverty and marital status (data available on request). Residents in intervention areas were matched to residents in control areas by using callipers of a width equal to 0.2 of a standard deviation of the logit of the propensity score (8). We used a 1:1 matching ratio (9).

We estimated standardised differences for all covariates before and after matching calculated as the absolute difference in sample means divided by the pooled standard deviation of the variable and multiplied by 100 to be expressed as a percentage. We used a standardised difference of 10% or more to be indicative of imbalance (10).

We estimated the effectiveness of targeted regeneration on inequalities in mental health by matching only those residents who received the intervention to control group residents based on their propensity scores to estimate the average causal effect for the exposed (11). This estimates the effect in residents in receipt of regeneration by matching the propensity scores of the 4,197 residents of areas which received regeneration to 4,197 participants who resided in areas that did not receive regeneration.

Web Appendix 2: Read codes used in the multiple imputation prediction model

Depression Diagnoses

Eu32	[X]Depressive episode
Eu320	[X]Mild depressive episode
Eu321	[X]Moderate depressive episode
Eu322	[X]Severe depressive episode without psychotic symptoms
Eu324	[X]Mild depression
Eu32y	[X]Other depressive episodes
Eu32z	[X]Depressive episode, unspecified
Eu33	[X]Recurrent depressive disorder
Eu330	[X]Recurrent depressive disorder, current episode mild
Eu331	[X]Recurrent depressive disorder, current episode moderate
Eu332	[X]Recurrent depressive disorder, current episode severe without psychotic symptoms
Eu334	[X]Recurrent depressive disorder, currently in remission
Eu33y	[X]Other recurrent depressive disorders
Eu33z	[X]Recurrent depressive disorder, unspecified
Eu341	[X]Dysthymia
E118	Seasonal affective disorder
E135	Agitated depression
E2B	Depressive disorder NEC
E2B1	Chronic depression
E291	Prolonged depressive reaction
E204	Neurotic depression reactive type

E2B0	Postviral depression
E112	Single major depressive episode
E1120	Single major depressive episode, unspecified
E1121	Single major depressive episode, mild
E1122	Single major depressive episode, moderate
E1123	Single major depressive episode, severe, without psychosis
E1125	Single major depressive episode, partial or unspecified remission
E1126	Single major depressive episode, in full remission
E112z	Single major depressive episode NOS
E113	Recurrent major depressive episode
E1130	Recurrent major depressive episodes, unspecified
E1131	Recurrent major depressive episodes, mild
E1132	Recurrent major depressive episodes, moderate
E1133	Recurrent major depressive episodes, severe, no psychosis
E1135	Recurrent major depressive episodes, partial/unspecified remission
E1136	Recurrent major depressive episodes, in full remission
E1137	Recurrent depression
E113z	Recurrent major depressive episode NOS

Anxiety Diagnoses

Eu41	[X]Other anxiety disorders
Eu410	[X]Panic disorder [episodic paroxysmal anxiety]
Eu411	[X]Generalized anxiety disorder
Eu413	[X]Other mixed anxiety disorders
Eu41y	[X]Other specified anxiety disorders

Eu41z	[X]Anxiety disorder, unspecified
E200	Anxiety states
E2000	Anxiety state unspecified
E2001	Panic disorder
E2002	Generalised anxiety disorder
E2004	Chronic anxiety
E2005	Recurrent anxiety
E200z	Anxiety state NOS

Mixed Depression and Anxiety Diagnoses

E2003	Anxiety with depression
Eu412	[X]Mixed anxiety and depressive disorder

Depression Symptoms

1B17	Depressed
1B1U	Symptoms of depression
1BQ	Loss of capacity for enjoyment
1BT	Depressed mood
1BU	Loss of hope for the future
2257	O/E – depressed

Anxiety Symptoms

1B13	Anxiousness
2258	O/E - anxious
1B12	Nerves, nervousness

R2y2	(D) Nervousness
2259	O/E Nervous

Drug Treatment Read Codes, Version 2 (algorithm included packet/bottle level)

Antidepressants

d71	Amitriptyline hydrochloride
d72	Butriptyline - discontinued
d73	Clomipramine hydrochloride
d74	Desipramine hydrochloride
d75	Dosulepin Hydrochloride
d76	Doxepin
d77	Imipramine hydrochloride
d78	Iprindole
d79	Lofepramine
d7a	Maprotiline hydrochloride
d7b	Mianserin hydrochloride
d7c	Nortriptyline
d7d	Protriptyline hydrochloride
d7e	Trazadone hydrochloride
d7f	Trimipramine
d7g	Viloxazine hydrochloride
d7h	Amoxapine
d81	Isocarboxazid
d84	Tranylcypromine
d85	Moclobemide
d91	Compound Antidepressants A-Z
da1	Flupentixol [Antidepressant]

da2	Tryptophan
da3	Fluvoxamine Maleate
da4	Fluoxetine hydrochloride
da5	Sertraline hydrochloride
da6	Paroxetine hydrochloride
da7	Venlafaxine
da9	Citalopram
daA	Reboxetine
daB	Mirtazapine
daC	Escitalopram
daD	Agomelatine
gde	Duloxetine

Hypnotics

d11	Chloral hydrate
d12	Clomethiazole edisylate (hypnotic)
d13	Dichloralphenazone - discontinued
d14	Flumtrazepam - discontinued
d15	Flurazepam
d16	Loprazolam
d17	Lormetazepam
d18	Nitrazepam
d1a	Temazepam (hynotic)
d1b	Triazolam - discontinued
d1c	Triclofos sodium
d1d	Zopiclone
d1f	Zolpidem

d1g	Zaleplon
d1h	Melatonin
d1i	Dexmedetomidine

Anxiolytics

d21	Diazepam
d22	Alprazolam
d23	Bromazepam
d24	Chlordiazepoxide
d25	Chlormezanone
d26	Clobazam
d27	Clorazepate dipotassium
d28	Hydroxyzine hcl (anxiolytic)
d29	Ketazolam - discontinued
d2a	Lorazepam (anxiolytic)
d2b	Medazepam - discontinued
d2c	Meprobamate
d2d	Oxazepam
d2f	Buspirone hydrocholoride
d2g	Flumazenil

Web Appendix 3: Sensitivity analyses

Web Tables 1–5 present the sensitivity analyses. Web Table 1 shows the estimated effectiveness of the intervention in a complete case sample. Web Table 2 shows the estimated effectiveness by the number of interventions in each theme of intervention. Web Table 3 the association between length of residence and change in mental health. Web Table 4 shows the change in mental health associated with residence in an intervention area after excluding migrants of intervention areas. Web Table 5 shows the full model adjusting for covariates rather than using propensity scores.

Web Table 3. Regression Coefficient (95% Confidence Interval) for Change in Mental Health Associated With Residence in an Intervention Area in a Complete-Case Sample (*n* = 4,426), Caerphilly County Borough, Wales, United Kingdom, 2001–2008

Model	Change in Mental Health	95% CI
Propensity scores [a, b]	1.33	0.27, 2.39

[a] Propensity scores were calculated using employment status, tenure, council tax valuation band, poverty status, and marital status.

[b] The Mental Health Inventory (MHI-5) comprised 5 items, and the total score was transformed to a 0 to 100 scale using a standard linear transformation (12).

Web Table 4. Regression Coefficient (95% Confidence Interval) for Change in Mental Health by Number of "Communities First" Projects Experienced in Each of the Themes (*n* = 8,394)

Type of Regeneration Project and No. of Projects Funded	Change in Mental Health [a, b]	95% CI	P-trend
Crime			
0	Referent	Referent	
1-33	1.46	0.10, 2.76	
34-63	1.09	-0.30, 2.48	0.59
Education			
0	Referent	Referent	
1-45	1.19	-0.26, 2.64	
46-120	-0.21	-2.26, 1.84	0.64
Health			
0	Referent	Referent	
1-24	1.11	-0.27, 2.49	
25-70	1.50	0.17, 2.83	0.61
Housing			
0	Referent	Referent	
1-173	1.52	0.14, 2.89	
174-335	1.08	-0.27, 2.43	0.57
Community			
0	Referent	Referent	
1-383	1.26	-0.11, 2.64	
384-887	1.34	-0.01, 2.69	0.62
Vocational training or business support			
0	Referent		
1-14	1.20	-0.08, 2.47	
15-25	1.58	0.04, 3.11	0.64

[a] Propensity scores were calculated using employment status, tenure, council tax valuation band, poverty status, and marital status.

[b] The Mental Health Inventory (MHI-5) comprised 5 items, and the total score was transformed to a 0 to 100 scale using a standard linear transformation (12).

Web Table 5. Regression Coefficient (95% Confidence Interval) for Change in Mental Health Associated With Length of Residence in an Intervention Area (*n* = 8,394)

Model	Length of Residence in an Intervention Area	Change in Mental Health	95% CI	*P*-trend
Propensity scores [a, b]	0 months (Referent)			
	0.2 to 41 months	-0.32	-4.95, 4.31	
	42 to 90 months	1.68	-0.97, 4.33	
	91 months (did not move in intervention area)	1.23	0.10, 2.36	0.05

[a] Propensity scores were calculated using employment status, tenure, council tax valuation band, poverty status, and marital status.

[b] The Mental Health Inventory (MHI-5) comprised 5 items, and the total score was transformed to a 0 to 100 scale using a standard linear transformation (12).

Web Table 6. Regression Coefficient (95% Confidence Interval) for Change in Mental Health Associated with Residence in an Intervention Area, Excluding Migrants of Intervention Areas (n = 7,871)

Model	Change in Mental Health	95% CI
Propensity score matching [a, b]	1.33	0.27, 2.39

[a] Propensity scores were calculated using employment status, tenure, council tax valuation band, poverty status, and marital status.

[b] The Mental Health Inventory (MHI-5) comprised 5 items, and the total score was transformed to a 0 to 100 scale using a standard linear transformation (12).

Web Table 7. Regression Coefficient (95% Confidence Interval) for Change in Mental Health Associated with Residence in an Intervention Area (*n* = 8,394), Caerphilly County Borough, Wales, United Kingdom, 2001–2008

Variable	Change in Mental Health Score	95% CI
Communities First area		
No	Referent	
Yes	1.55	0.40, 2.71
Employment status		
Employed	Referent	
Unemployed	-2.88	-7.24, 1.49
Student	-2.02	-7.18, 3.15
Homemaker or caregiver	-1.28	-4.00, 1.45
Permanently sick or disabled	6.90	4.78, 9.01
Retired	3.92	2.10, 5.73
Tenure		
Not an owner occupier	Referent	
Owner occupier	1.76	0.10, 3.40
Council tax valuation band		
A (lowest property value)	Referent	
B	2.09	0.45, 3.71
C	3.83	1.58, 5.20
D	2.45	-0.34,5.59
E	4.69	1.11, 8.59
F-H (highest)	4.43	-0.83, 9.12
Poverty status		
In poverty	Referent	
Not in poverty	1.49	-0.14, 3.12
Marital status		
Married/cohabiting	Referent	
Single	-1.93	-3.76, -0.10
Divorced or separated	0.85	-4.13, 5.85
Widowed	1.32	-3.17, 5.82

References

1. Hayes V, Morris J, Wolfe C, et al. The SF-36 Health Survey Questionnaire: Is it suitable for use with older adults? *Age Ageing*. 1995;24(2):120–125.

2. Hill S, Harries U, Popay J. Is the short form 36 (SF-36) suitable for routine health outcomes assessment in health care for older people? Evidence from preliminary work in community based health services in England. *J Epidemiol Community Health*. 1996;50(1):94–98.

3. Austin PC. An introduction to propensity score methods for reducing the effects of confounding in observational studies. *Multivariate Behavioral Res*. 2011;46(3):399–424.

4. Austin PC. A tutorial and case study in propensity score analysis: an application to estimating the effect of in-hospital smoking cessation counseling on mortality. *Multivariate Behavioral Res*. 2011;46(1):119–151.

5. Haukoos JS, Lewis RJ. The propensity score. *JAMA*. 2015;314(15):1637–1638.

6. Rosenbaum PR, Rubin DB. The central role of the propensity score in observational studies for causal effects. *Biometrika*. 1983;70(1):41–55.

7. Rubin DB, Thomas N. Matching using estimated propensity scores: relating theory to practice. *Biometrics*. 1996;249–264.

8. Austin PC. Optimal caliper widths for propensity-score matching when estimating differences in means and differences in proportions in observational studies. *Pharmaceutical Stat*. 2011;10(2):150–161.

9. Austin PC. Comparing paired vs nonpaired statistical methods of analyses when making inferences about absolute risk reductions in propensity-score matched samples. *Stat Med*. 2011;30(11):1292–1301.

10. Austin PC. Balance diagnostics for comparing the distribution of baseline covariates between treatment groups in propensity-score matched samples. *Stat Med*. 2009;28(25):3083–3107.

11. Williamson E, Morley R, Lucas A, et al. Propensity scores: from naive enthusiasm to intuitive understanding. *Stat Methods Med Res*. 2012;21(3):273–293.

12. Kelly MJ, Dunstan FD, Lloyd K, et al. Evaluating cutpoints for the MHI-5 and MCS using the GHQ-12: a comparison of five different methods. *BMC Psychiatry*. 2008;8(1):10.
